# Does Bright Light Counteract the Post-lunch Dip in Subjective States and Cognitive Performance Among Undergraduate Students?

**DOI:** 10.3389/fpubh.2021.652849

**Published:** 2021-06-07

**Authors:** Ying Zhou, Qingwei Chen, Xue Luo, Le Li, Taotao Ru, Guofu Zhou

**Affiliations:** ^1^Lab of Lighting and Physio-Psychological Health, National Center for International Research on Green Optoelectronics, South China Normal University, Guangzhou, China; ^2^Guangdong Provincial Key Laboratory of Optical Information Materials and Technology & Institute of Electronic Paper Displays, South China Academy of Advanced Optoelectronics, South China Normal University, Guangzhou, China; ^3^School of Psychology, South China Normal University, Guangzhou, China

**Keywords:** light, post-lunch dip, alertness, mood, cognitive function, sleep restriction

## Abstract

The post-lunch dip in alertness and performance was widely experienced during the early afternoon. Taking a short nap was documented as a practical strategy for habitual nappers to counteract the decline of alertness and performance. Yet, it remains unknown whether bright light exposure in the early afternoon working hours could alleviate the performance deficits caused by a post-lunch nap loss for habitual nappers. Seventeen undergraduate students who had a long-term habit of taking a post-lunch nap were assigned to three interventions: (1) a short nap + normal indoor light (100 lx, 4,000 K at eye level); (2) no nap + normal indoor light, and (3) no nap + blue-enriched bright light (1,000 lx, 6,500 K at eye level), in which subjective alertness (Karolinska Sleepiness Scale, KSS), mood (Positive and Negative Affect Schedule, PANAS), and task performance in sustained attention (psychomotor vigilance test, PVT), response inhibition (go/no-go task), and working memory (paced visual serial addition test, PVSAT) were measured. Results showed that a post-lunch nap deprivation significantly increased subjective sleepiness and negative mood and impaired performance in PVT and PVSAT, while exposure to bright blue-enriched white light vs. normal indoor light in the early afternoon significantly relieved such negative effects on mood, sleepiness, and performance in PVSAT; subjective positive mood and performance in PVT and go/no-go task remained unaffected with light intervention. These findings suggested that bright blue-enriched white light exposure could be a potential strategy for those who are suffering from drowsiness and low working memory following a habitual midday nap loss.

## Introduction

Lack of alertness or higher sleepiness is experienced not only during nighttime but also shortly in early afternoon (14:00–16:00, the so-called “post-lunch dip”), which was addressed as the circadian-driven daytime vigilance could not be sufficiently robust to counteract the homeostatic sleep urge (1, 2). The post-lunch dip-induced decrements in alertness and behavioral performance are a widespread problem in the workplace, which were considered as the major causes of serious work-related errors and accidents, such as in car driving, air traffic control, and intensive medical care (3–6). Seeking for a practical strategy to counteract the decline of alertness and performance and to even enhance afternoon function would be of great value to students or employees at work.

In some countries, such as in China, most university students are used to take a short post-lunch nap during working days. Multiple empirical studies have demonstrated that people taking a brief nap (20–40 min) would promote afternoon function including vigilance, sustained attention, executive functions, logic reasoning, memory, and learning performance (5, 7–12), whereas a lost opportunity of midday nap would lead to various impaired effects on alertness, mood, and performance ability, especially for those who habitually takes a nap (7, 9–11).

In addition to taking a brief nap, light can induce acute alerting effects (13–15) and benefit affective state (16–18) and cognitive functioning (19–21) via a non-image-forming (NIF) pathway, thus holding the potentials to counteract the post-lunch dip in subjective state and task performance. The current literatures have—although not always—demonstrated that exposure to bright white light or bright blue-enriched white light or combining that with a short nap during post-lunch period would significantly enhance subsequent alertness and performance (4, 5, 8, 22). For instance, Kaida et al. (23) reported that exposure to natural light (around 3,260 lx vs. dim light) for 30 min during post-lunch period decreased afternoon sleepiness but did not affect psychomotor vigilance task performance. They further investigated the effects of bright light (>2,000 lx) and an afternoon nap (20 min) on a working memory task (5) and a visual search and implicit learning task (8). The findings showed that performance on executive coordination and visual search was improved with an afternoon nap but not with bright light. By contrast, Slama et al. (9) found that bright light (2,000 lx) exposure during lunchtime (13:30–14:00) enhanced cognitive flexibility in the early afternoon. One laboratory study by Baek and Min (22) demonstrated that exposure to blue-enriched (33% peak wavelength, 451 nm) compared to darkness and white light in the early afternoon (14:00–15:00) significantly reduced the EEG alpha activity and increased reaction speed on sustained attention task. Whereas, Askaripoor et al. (24) also found blue-enriched white light (12,000 K, 500 lx) would improve physiological alertness as measured by lower alpha-band power than normal white light (4,000 K, 500 lx) did, but failed to improve subjective alertness and performance on sustained attention, divided attention, inhibitory capacity, and working memory.

The above inconstant findings could be resulted by several factors, except the differences in light properties and study paradigms; the type of task dependency in the direction and magnitude of NIF effects of light was also established in previous studies on diurnal cognitive effects of bright vs. dim light (25–30). For instance, Smolders et al. (25) revealed that bright vs. dim (1,000 vs. 165 lx) white light during working hours improved vigilance performance but impaired working memory performance. One recent study by Ru et al. (30) showed that exposure to high (1,000 lx) vs. low (100 lx) illuminance selectively improved task performance on response inhibition and conflict monitoring but not on sustained attention or working memory. Yet, it remains largely unknown whether the intervention effects of bright light vs. normal indoor light exposure on post-lunch dip in performance would be task-specific.

Moreover, as we mentioned before, the loss of opportunities to take a nap at noon time would result in deleterious effects on alertness and cognitive performance. As in most of these studies participants were non-habitual nappers, it is difficult to determine whether bright light exposure during the post-lunch dip period could alleviate the deficits of subjective state and task performance caused by post-lunch nap deprivation for university students who were habitual nappers. A line of research about light's countermeasures for night sleep deprivation or sleep restriction might provide us some inspiration to the current investigation. For example, Wright et al. (31) found bright light (2,500 lx) could enhance both nighttime performance and morning performance during extended sleep deprivation. A following study by Viola et al. (32) reported the beneficial role of bright light on subjective and objective alertness and working memory performance after one night of sleep deprivation.

Together, the above findings suggested the effectiveness of bright white light or blue-enriched white light to overcome the decline of alertness and performance under the condition of night sleep deprivation. Yet, the potential benefits of bright light intervention following daytime nap deprivation among habitual nappers had been scarcely investigated. Thus, the current study was conducted to firstly explore the effects of blue-enriched bright light (1,000 lx, 6,500 K, at eye level; BL) intervention vs. normal indoor light (100 lx, 4,000 K, at eye level; NL) on alertness, mood, and task performance during the post-lunch dip period among healthy university students who were habitual nappers. Secondly, the current study aims to investigate whether the effects of light intervention on task performance are dependent on the type of task.

## Methods

### Participants and Screening Procedure

Seventeen university students (11 females, mean age = 20.47 years, range 18–23 years) with a long-term habit of an afternoon nap (between 13:00 and 14:00) participated in the current study. All participants provided informed written consent at their first arrival in the laboratory. All of them were required to complete the online questionnaires 1 week before the laboratory study and extensively screened for mental and physical health according their response on the General Health Questionnaire-20 (33–35) and three items (“Are you suffering with any physical ailments”; “Have you had a cold, headache or other somatic symptoms in the past week”; and “Have you taken any medication in the past week for any physical ailments”). None of them (1) habitually napped <30 min and >50 min; (2) were smokers and consuming medications or drugs; (3) traveled to a different time zone or shift work in the last 3 months; (4) were extremely late or early chronotype on the Munich Chronotype Questionnaire (36, 37); (5) have a night sleep <7 h and >9 h; (6) have a body mass index <20 and <25; (7) scored >5 on the Pittsburgh Sleep Quality Index (38, 39); and (8) scored >8 on the Beck Depression Inventory-II (40, 41).

### Experiment Design

A one-factor light intervention within-subject design was employed in the current study. Participants were assigned to three interventions: (1) a short nap and normal indoor light (nap + NL) condition, (2) no nap and normal indoor light (ND + NL) condition, and (3) no nap and blue-enriched bright light (ND + BL) condition. The full experiment session lasted for three non-consecutive days with an interval of at least 3 days to minimize any carry-over effects, and three interventions were counterbalanced with Latin square order.

### Procedure

**Figure 1:**
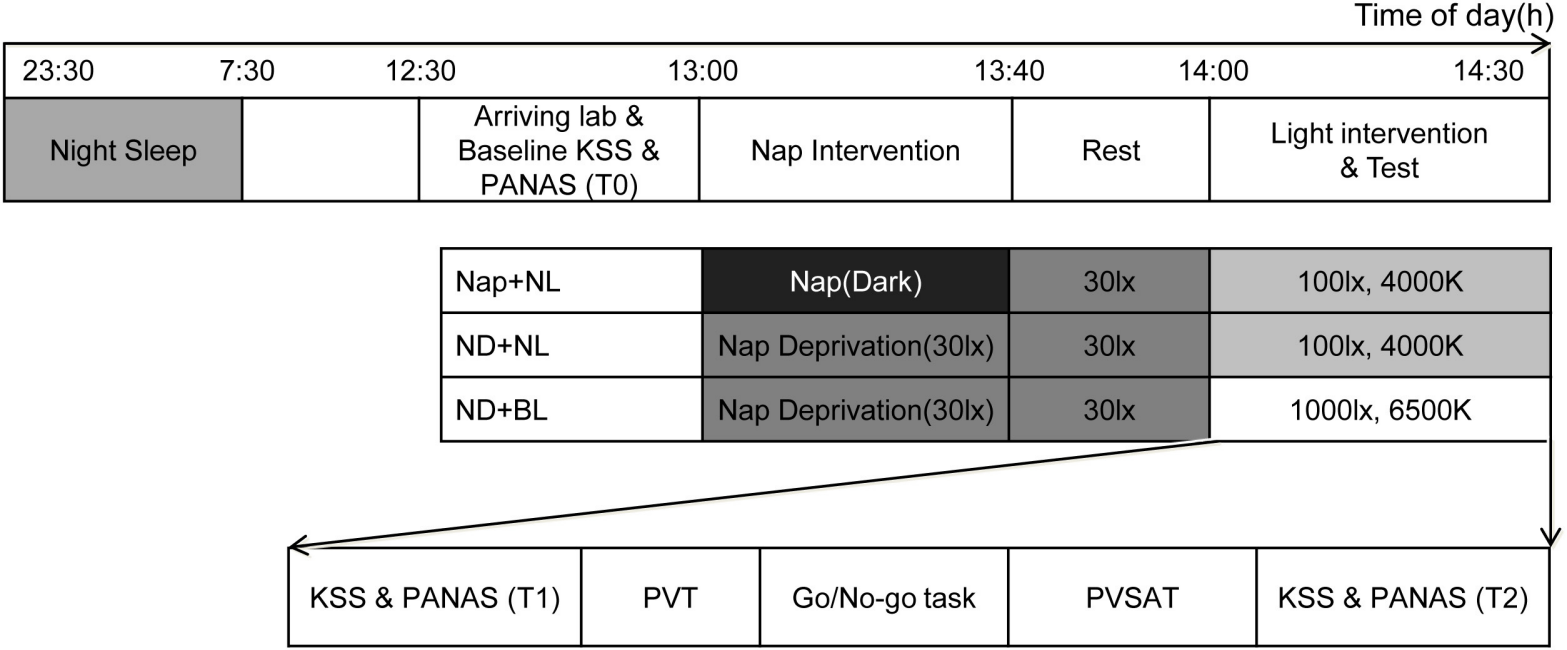
A schematic representation of the overall study protocol. The sleep–wake schedule was one example for participants who slept at 23:30 p.m. and woke up at 7:30 a.m. The order of tasks was performed pseudo randomly and remained the same on three experimental days. NL, normal light; BL, blue-enriched bright light; KSS, Karolinska Sleepiness Scale; PANAS, Positive and Negative Affect Schedule; PVT, Psychomotor Vigilance Test; PVSAT, Paced Visual Serial Addition Test.

Figure 1 represents the time frame of one experimental session. Participants were informed to comply with their regular night sleep schedule and abstain from drinking beverages containing alcohol and caffeine on experiment days. The study was conducted in agreement with the regulations of the Ethics Committee on Research involving Humans at South China Normal University.

All participants were required to sleep according to their daily sleep–wake schedule (usually sleep between 23:30 p.m. and 7:30 a.m.) at preceding night on experiment day, and a time difference of 30 min in sleep and wake time was allowable. Participants were asked to finish their lunch before 12:30 p.m. and arrive in the sleep laboratory at 12:40 p.m. Their baseline sleepiness was measured with the Karolinska Sleepiness Scale (KSS), and mood was assessed with the Positive Affect and Negative Affect Schedule around 13:00 p.m. The nap intervention session was started at 13:00 p.m. in which participants were assigned to either take a 40-min nap (nearly dark) or stay awake (under 30 lx, 4,000 K light at eye level) for 40 min in the laboratory. After that, all participants received a 20-min opportunity for free activities in the laboratory to minimize any potential influence of sleep inertia. The light intervention session was started at 14:00 p.m. including questionnaires on subjective alertness and mood and three computerized cognitive tests. At end of the light intervention session, participants' sleepiness and mood were measured again.

**Figure 2:**
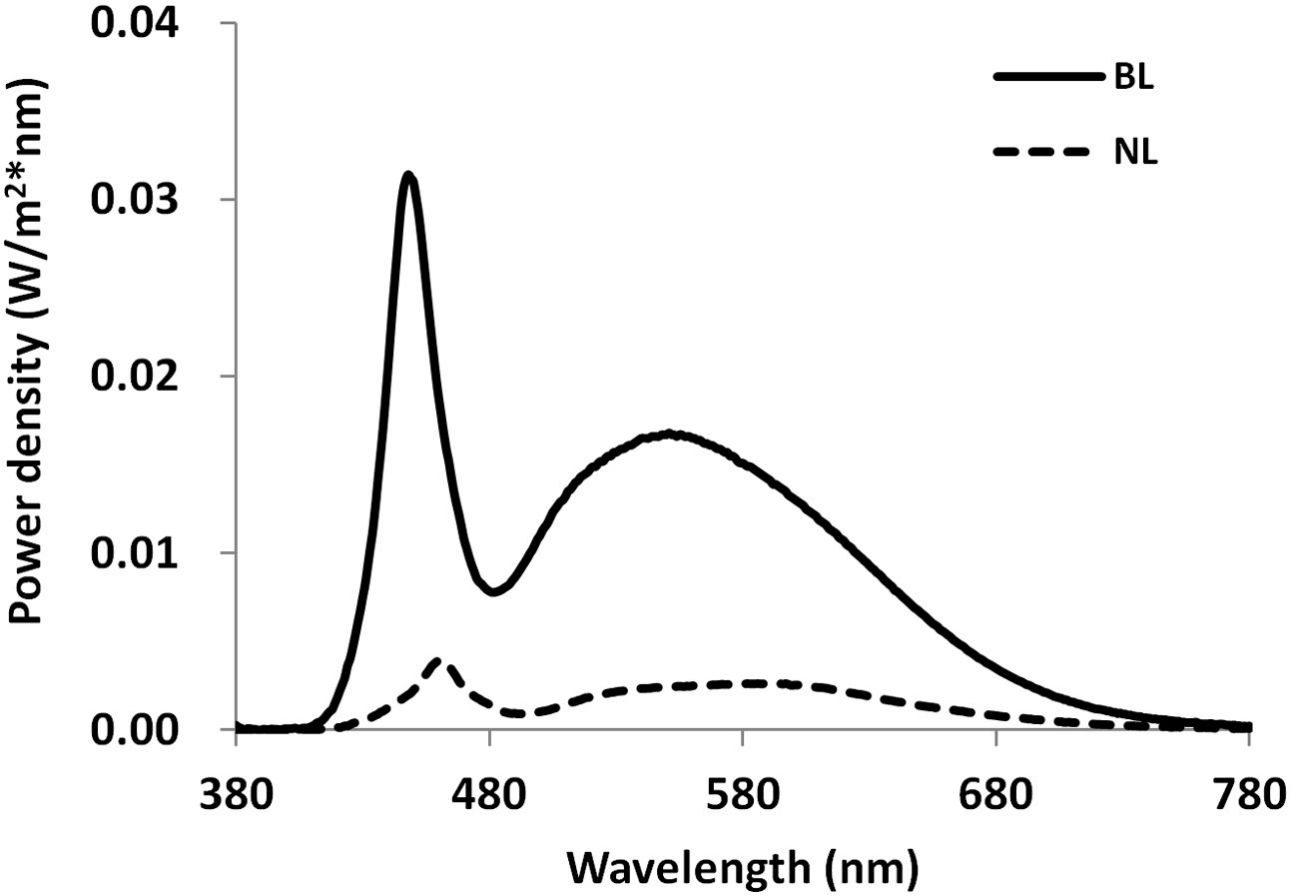
Spectral power distributions measured at eye level for normal light (100 lx, 4,000 K) and blue-enriched bright light (1,000 lx, 6,500 K) conditions. NL, normal light; BL, blue-enriched bright light.

The lab room where the light intervention was conducted was a simulated office environment with the size of 3.6 by 3.6 m. Four separate workstations with one pure white desk (1.2 by 0.8 m) and one blank chair were created. The room was equipped with six ceiling-mounted luminaires of 1.2 by 0.8 m containing three LED tubes (T8-28W) and three additional ceiling-mounted luminaires of 1.2 by 0.6 m containing LED tubes (T8-28W). During the lighting intervention phase, lighting was set to either 1,000 lx at 6,500 K at eye level in ND + BL condition or 100 lx at 4,000 K at eye level in the nap + NL and ND + NL condition. Using a calibrated spectroradiometer (JETI Specbos1201), the illuminance level and spectral power distribution (SPD) were measured at the eye level of participants. Figure 2 shows the SPDs for the normal light condition and the blue-enriched bright light condition. The effective irradiances for each retina photoreceptors of two light conditions are shown in Table 1 (42, 43).

**Table 1:** Spectrally weighted α-opic illuminance levels for normal light condition and blue-enriched bright light condition.

		**α-Opic Lx value**	
	**λ_**max**_ (nm)**	**100 lx, 4,000 K**	**1,000 lx, 6,500 K**
Lx		111	1,040
CCT		4,430	6,666
Melanopsin	480.0	83	998
S-cone	419.0	80	1,081
M-cone	530.8	102	1,044
L-cone	558.4	108	1,003
Rods	496.3	89	1,026

*The five α-opic irradiances were determined by using the calculation toolbox developed by Lucas et al. (42)*.

### Measurements

#### Subjective Sleepiness and Mood

The KSS was used to assess subjective sleepiness ranging from (1) extremely alert to (9) extremely sleepy (44). Positive mood and negative mood were evaluated using the Positive Affective and Negative Affect Schedule (PANAS) ranging from (1) extremely light to (5) extremely strong (45, 46).

#### Psychomotor Vigilance Test

A 10-min auditory Psychomotor Vigilance Test (PVT) (47) was used to assess objective alertness and sustained attention. During the task, participants were required to keep their dominant hand on the space bar and respond as fast as possible after hearing a 1,000-Hz beep, which was presented using a random inter-stimuli interval between 1,000 and 9,000 ms. Lapse probability and average reaction speed for overall trials and 10% fastest and 10% slowest trials were computed separately.

#### Go/No-Go Task

A visual go/no-go task measured the capacity for response inhibition (48). In this task, participants were required to press the key button within 1,000 ms if the letter “M” (go trials) was shown on the screen and withhold response to letter “W” (no-go trials). The interval between trials was randomized between 1,000 and 1,500 ms and a total of 70% of “M” letters was presented in a random sequence. The ratio of the correct number of response to “go” and withdraw response to “no-go” trials and speed for correct response to “go” trials were investigated separately.

#### Paced Visual Serial Addition Test

Paced Visual Serial Addition Test (PVSAT) is an additional task that engages with the executive aspect of working memory (49). Single digits (1–15) appeared on screen, and each needed to be added to the digit that preceded it. The resulting answer was keyed in with numerical keyboard (“sum” of adjacent pairs, not a total across all digits presented). Digits were presented for 1,000 ms, and then responses were allowed within an additional 500 ms maximum or disappear until response was detected, which is followed by a 1,000-ms interval. Accuracy and reaction speed for correct response were measured separately.

### Data Analysis

The measure of positive mood and negative mood is determined by averaging the scores of 9 positive adjectives and 9 negative adjectives, respectively. To increase normality, all reaction time (RT) data were inverted before further analysis to improve the normality of data distribution. Reaction speed in incorrect trials and outliers (more than three standard deviations from the mean) were removed for all tasks before further analysis. For the PVT data, lapses were determined as the total number of trials with RTs longer than 500 ms and trials without a response. Lapse probability was calculated as the number of lapses divided by the total number of valid trials.

SPSS 19.0 (IBM, USA) was used for all the analysis. A repeated measures analysis of variance (ANOVA) with factor intervention (nap + NL, ND + NL, and ND + BL) was employed on all dependent variables. Since subjective alertness and mood were assessed multiple times during the intervention, testing time (T0, T1, and T2) was added as a within-participant factor in the analysis. The Greenhouse–Geisser adjustment was applied when necessary. The Bonferroni correction was employed for the *post-hoc* analysis.

## Results

### Sleepiness

**Figure 3:**
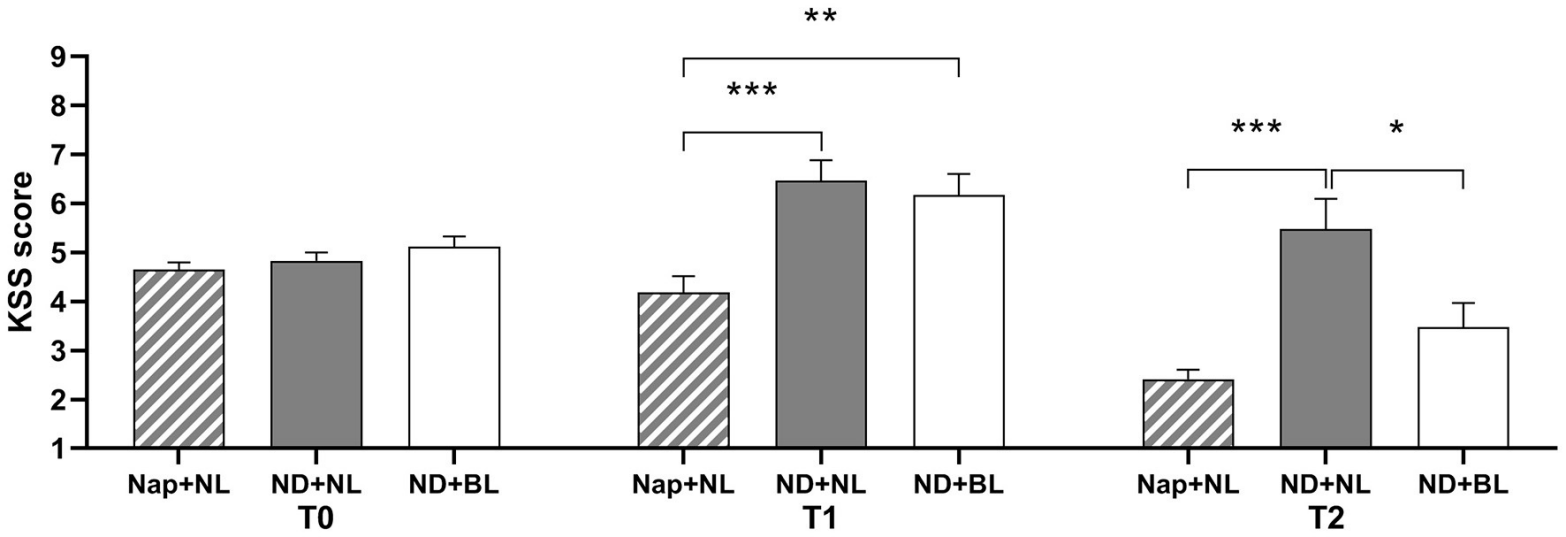
Subjective sleepiness assessed by KSS. Error bars represent one standard error of the mean. ***p < 0.001, **p < 0.01, *p < 0.05.

The ANOVA of subjective sleepiness revealed significant main effect for intervention and test time and a significant interaction between intervention and test time (see Table 2 and Figure 3). *Post-hoc* test indicated there was no significant difference in subjective sleepiness across three intervention conditions at baseline. Participants' sleepiness was significantly lower in the nap + NL condition (3.00 ± 0.21) than the ND + NL (5.82 ± 0.53, *p* < 0.001) and ND + BL conditions (4.71 ± 0.39, *p* = 0.007). At the end of nap deprivation (T1), sleepiness did not differ between the ND + NL and ND + BL conditions at T1 (*p* > 0.05). However, participants felt significantly less sleepy both in the nap + NL (2.41 ± 0.19, *p* < 0.001) condition and the ND + BL condition (3.47 ± 0.49, *p* = 0.043) than in the ND + NL (5.47 ± 0.63) condition. At the end of the light intervention (T2), no significant difference in sleepiness was found between the ND + BL condition and the nap + NL condition (*p* > 0.05).

**Table 2:** Results of ANOVA for subjective measurements.

	**Intervention**				**Test time**				**Intervention × Test time**			
	***F***	***df***	***p***	***η^2^***	***F***	***df***	***p***	***η^2^***	***F***	***df***	***p***	***η^2^***
Sleepiness	**17.76**	**(2,15)**	**<0.001**	**0.53**	**10.46**	**(2,15)**	**<0.001**	**0.40**	**7.23**	**(4,13)**	**<0.001**	**0.31**
Mood												
Positive	**3.85**	**(2,15)**	**0.032**	**0.19**	1.72	(2,15)	0.195	0.10	0.69	(4,13)	0.605	0.04
Negative	**8.41**	**(2,15)**	**0.001**	**0.34**	0.66	(2,15)	0.525	0.04	**3.37**	**(4,13)**	**0.014**	**0.17**

*Significant differences are indicated in bold*.

### Mood

**Figure 4:**
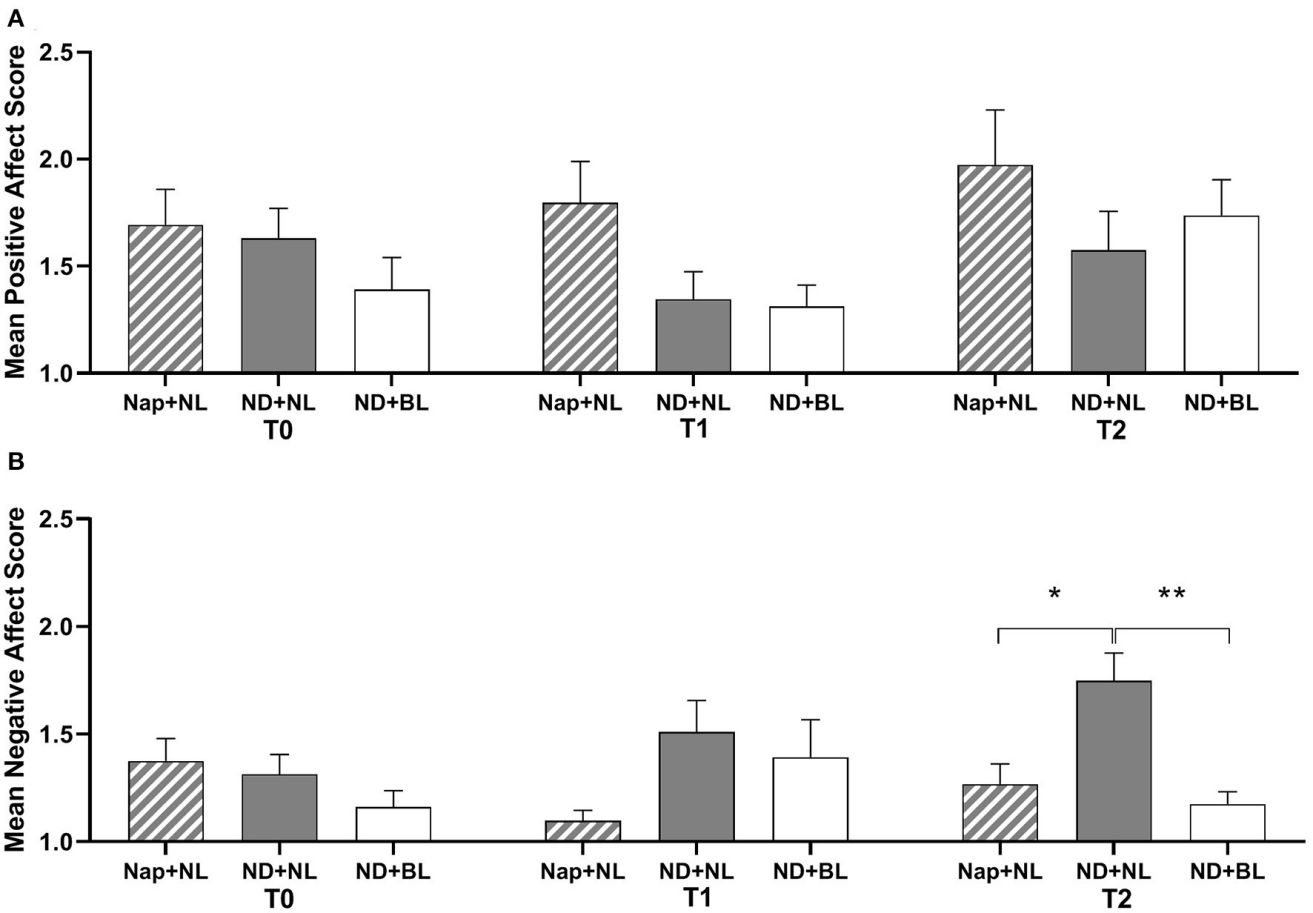
Subjective mood assessed by PANAS. **(A)** Positive mood; **(B)** negative mood. Error bars represent one standard error of the mean. **p < 0.01, *p < 0.05.

The results of subjective positive mood revealed a significant main effect of intervention. However, the *post-hoc* analysis indicated no significant differences among the three interventions after Bonferroni correction (see Figure 4A). Besides that, neither significant main effects of test time nor interaction effect between intervention and test time were revealed for positive mood (see Table 2). For negative mood, both the intervention and the interaction between intervention and test time yielded a significant effect (see Table 2). *Post-hoc* test showed that participants experienced more negative mood in the ND + NL (1.75 ± 0.13) than in the nap + NL condition (1.27 ± 0.09, *p* = 0.039) and ND + BL condition (1.18 ± 0.06, *p* = 0.003), which only occurred at the end of the light intervention (T2). No significant difference was revealed between the nap + NL and ND + BL conditions at T2 (Figure 4B).

### PVT

**Figure 5:**
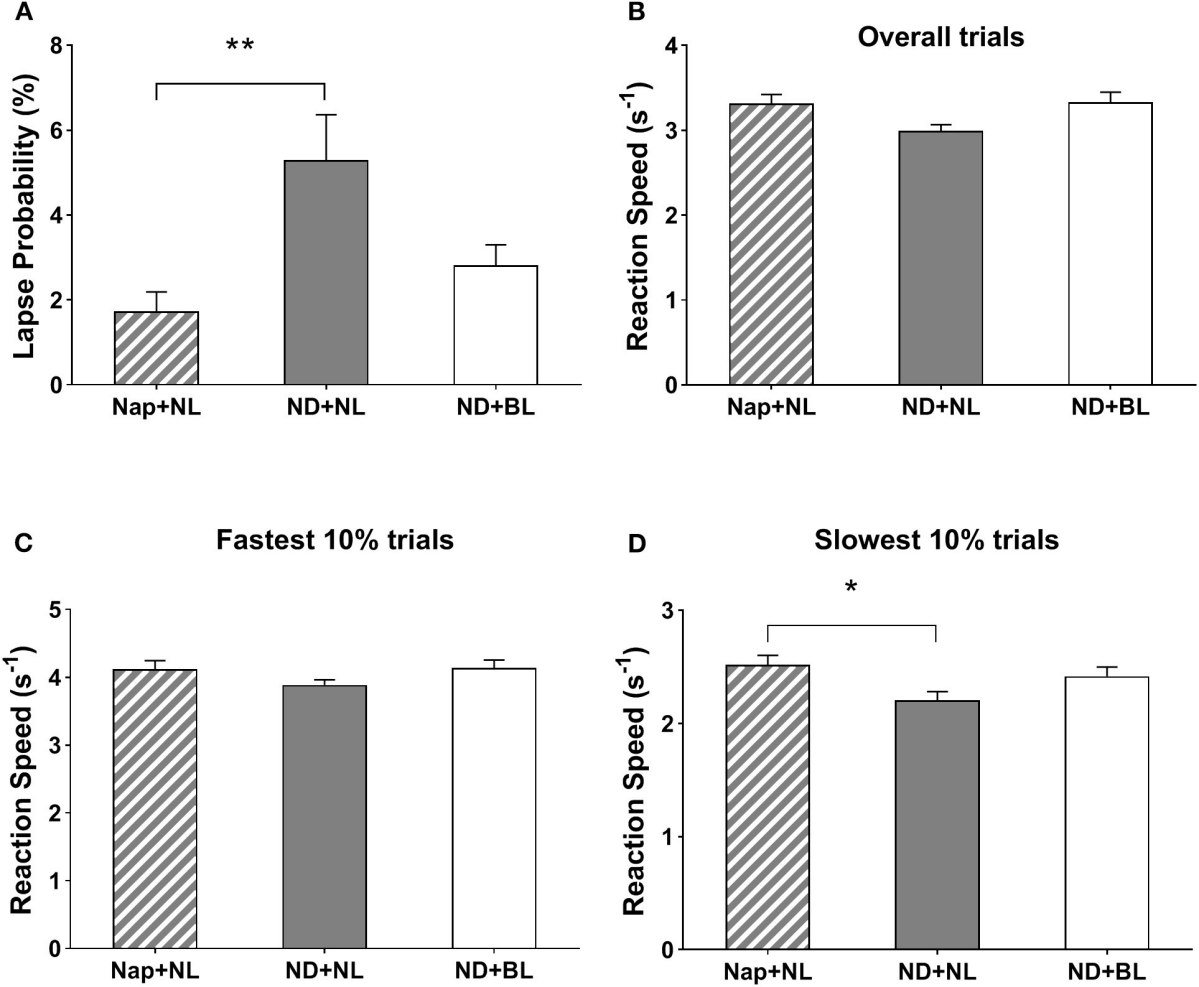
PVT performance. **(A)** Lapse probability; **(B)** average reaction speed for all valid trials; **(C)** average reaction speed for the fastest 10% of the trials; and **(D)** average reaction speed for the slowest 10% of the trials. Error bars represent one standard error of the mean. **p < 0.01, *p < 0.05.

The intervention yielded a significant effect on lapse probability, overall reaction speed, and 10% slowest reaction speed in PVT [*F*_(2,15)_ = 8.17, *p* = 0.001, η^2^ = 0.34; *F*_(2,15)_ = 3.92, *p* = 0.03, η^2^ = 0.20; *F*_(2,15)_ = 4.69, *p* = 0.02, η^2^ = 0.23]. The *post-hoc* test indicated lapse probability was less in the nap + NL (1.72 ± 0.46%) vs. ND + NL conditions (5.30 ± 1.06%, *p* = 0.009). Lapse probability in the ND + BL (2.82 ± 0.48%) did not significantly differ with the other two conditions. Similarly, participants' reaction speed for the 10% slowest trials was significantly lower in the nap + NL (2.51 ± 0.08) vs. ND + NL condition (2.20 ± 0.08, *p* = 0.047). No significant differences were found for the 10% slowest reaction speed (ND + BL: 2.41 ± 0.08). As for the overall reaction speed, the differences among conditions did not reach statistical significance after Bonferroni correction. Intervention did not yield a significant main effect on the 10% fastest reaction speed in PVT [*F*_(2,15)_ = 2.26, *p* = 0.12, η^2^ = 0.12] (see Figure 5).

### Go/No-Go Task

**Figure 6:**
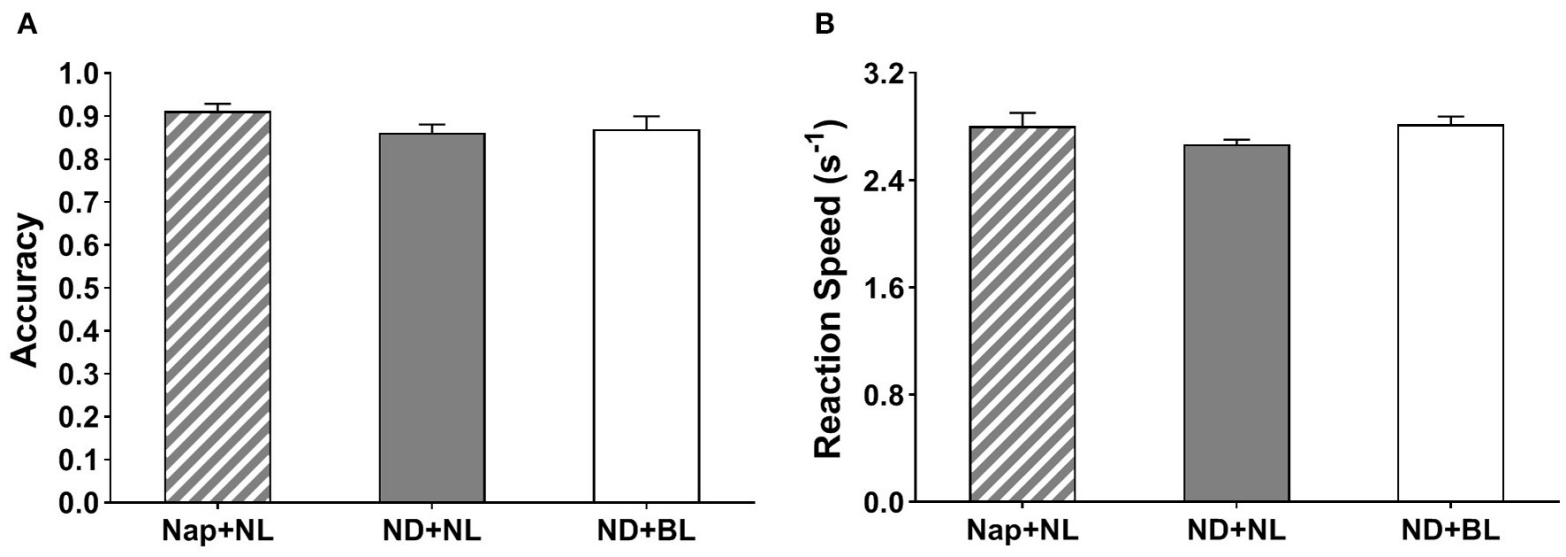
Go/no-go task performance. **(A)** Accuracy; **(B)** average reaction speed. Error bars represent one standard error of the mean.

Neither the reaction speed nor accuracy in the go/no-go task revealed a significant main effect of intervention [*F*_(2,15)_ = 2.00, *p* = 0.15, η^2^ = 0.11; *F*_(2,15)_ = 2.55, *p* = 0.12, η^2^ = 0.14] (see Figure 6).

### PVSAT

**Figure 7:**
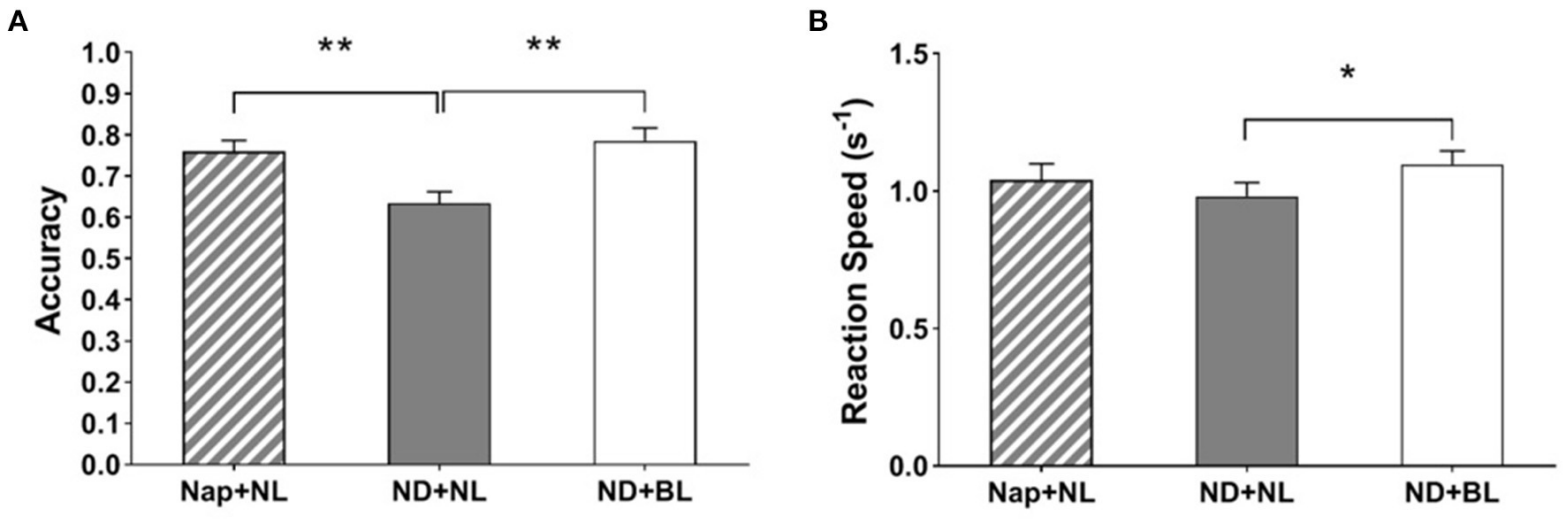
PVSAT performance. **(A)** Accuracy; **(B)** average reaction speed. Error bars represent one standard error of the mean. **p < 0.01, *p < 0.05.

Both accuracy and reaction speed in PVSAT showed a significant main effect of intervention [*F*_(2,15)_ = 11.03, *p* < 0.001, η^2^ = 0.41; *F*_(2,15)_ = 4.50, *p* = 0.02, η^2^ = 0.22]. The *post-hoc* comparisons indicated that participants had higher accuracy in the nap + NL condition (0.76 ± 0.03) and the ND + BL condition (0.78 ± 0.03) than in the ND + NL condition (0.63 ± 0.03, both *p* < 0.01). Whereas, the reaction speed was only significantly higher in the ND + BL condition (1.10 ± 0.05) than in the ND + NL condition (0.98 ± 0.05, *p* = 0.023) (see Figure 7). No other significant differences were revealed (nap + NL: 1.04 ± 0.25).

## Discussion

The current study was conducted in an effort for the first time to explore the effects of bright indoor light on alertness, mood, and task performance during the post-lunch dip period among healthy university students who were midday nap deprived, extending previous studies about the intervention effects of light on performance deficits caused by nighttime sleep restriction and post-lunch dip in non-habitual nappers (9, 22, 24, 31, 32, 50). The current findings suggested that bright blue-enriched white light (1,000 lx, 6,500 K, at eye level) vs. normal indoor light exposure (100 lx, 4,000 K, at eye level) in the early afternoon could hold the potential to counteract the post-lunch dip in alertness, negative mood, and working memory performance among habitual nappers.

For subjective sleepiness, the current findings revealed that participants' subjective sleepiness was significantly higher in the nap deprivation with normal light condition vs. nap with normal light condition at the end of nap manipulation and also sustained at the end of test session. These findings were in line with previous studies suggesting a brief nap during post-lunch dip period would significantly decrease subjective sleepiness (4, 51) and increase physical arousal in terms of lower theta and alpha power (52, 53). Meanwhile, the current findings revealed that exposure to bright blue-enriched white light in the early afternoon could prevent the decline of subjective alertness induced by a post-lunch nap deprivation. Participants in the nap-deprived with bright blue-enriched white light condition vs. nap deprivation with normal light condition experienced significantly lower sleepiness and that was comparable to the nap with normal light condition. These findings were partially in line with those of previous studies suggesting either positive or null effects of bright vs. dim light on subjective assessment of alertness (25, 28, 29, 54–56). Even though most of the previous studies [62%, according to Souman et al. (15)] reported significant alerting responses to bright vs. dim light during daytime, several studies even did not (28, 29, 54). In addition to the differences in light properties, the individual's prior state to the light manipulation would also moderate the light-induced NIF effects. One study by Smolders and de Kort (57) suggested a state-dependent alerting effect of high-intensity level during daytime, with alerting effects being more pronounced when participants experienced more mental fatigue. It thus would be speculated that participants would be more sensitive to bright light exposure as they experienced higher fatigue and sleepiness following a habitual post-lunch nap deprivation.

For subjective mood, the current findings revealed that a habitual post-lunch nap deprivation impaired habitual nappers' subjective mood, with higher experienced negative mood in no nap with normal light condition vs. nap with normal light condition, while participants' positive mood remained unaffected with the nap deprivation. Effects of a nap deprivation on mood were scarcely investigated in previous studies, except one earlier study by Caldwell and Caldwell (58) suggesting beneficial effects of a post-lunch nap on mood. One recent study by Qian et al. (59) reported that participants who took a short midday nap felt happier than those who stayed awake during post-lunch dip period. Similar to the case of subjective sleepiness, the current study revealed exposure to bright blue-enriched white light in the early afternoon would relieve habitual nappers' negative mood stimulated by nap deprivation. Participants' negative mood was significantly lower in the no-nap with bright blue-enriched white light condition vs. the no-nap with normal light condition, suggesting that bright white light might be a more desirable option for mood stabilizer than normal office light level in the context of post-lunch nap deprivation. These limited effects on mood replicated previous studies (30, 60, 61), while contradicted another one (62). Again, participants employed in the study by Lan et al. (62) were all well-rested, which were quite different with those in the current study. An alternative explanation is that a habitual midday napper might experience higher mental fatigue resulted by a nap deprivation (63, 64), which would be easily alleviated to some extent by the ambient bright light exposure.

Regarding the task performance, the previous studies had revealed no consistent pattern of a post-lunch nap on task performance varied in different cognitive domains (5, 9, 10, 23, 59). The current findings even revealed an indicator and type of task-dependent impairment of nap deprivation on cognitive performance. To be specific, participants in the no-nap with normal light condition vs. nap with normal light condition increased lapse probability, lowered 10% slowest reaction speed in PVT assessing sustained attention, and lowered accuracy in PVSAT assessing working memory, whereas performance in response inhibition as assessed with the go/no-go task remained unaffected with nap deprivation. In contrast to the effects of light intervention on subjective states, the current findings did not reveal consistent restorative effects of blue-enriched white light on task performance across cognitive domains. For the sustained attention, exposure to bright blue-enriched white light vs. normal light following a habitual nap deprivation did not significantly improve the 10% slowest reaction speed and lower the lapse probability in PVT. As we mentioned above, the bright light-induced alerting effects were not always reported in previous studies [see review (65)], whereas several recent studies even failed to reveal the alerting effects of bright vs. dim light during daytime among those who did not experience any night sleep or daytime nap restrictions (30, 66–68). These inconsistencies might be caused by the differences in the light properties and timing of light intervention. For instance, Baek and Min (22) demonstrated that exposure to blue-enriched light compared to darkness and white light in the early afternoon significantly increased physical arousal and reaction speed on sustained attention task. However, blue light (33% peak wavelength, 451 nm) was employed in their study and bright white light (6,500 K, 1,000 lx) was employed in the current study. Besides that, the time of day was documented as a potential factor moderating the magnitude and direction of diurnal NIF effects of light, with more pronounced effects of bright vs. dim light on subjective state and sustained attention in the morning vs. afternoon (25, 69). The current study was conducted only in the afternoon leading less possible to test the moderator role of time of day.

Besides, the current findings revealed that exposure to bright blue-enriched white light in the early afternoon selectively enhanced performance in relatively higher executive function. Participants' performance in PVSAT was significantly enhanced in no-nap with bright blue-enriched white light condition vs. no-nap with normal indoor light condition, whereas performance in the go/no-go task remained unaffected with light intervention. Previous studies—however—revealed inconclusive effects, including positive, null, and even negative effects of daytime bright light on response inhibition tasks (24, 27, 57, 70), as well as on working memory tasks (24, 27–29, 57, 71). One recent study by Askaripoor et al. (24) reported blue-enriched white light (12,000 K, 500 lx) vs. normal white light (4,000 K, 500 lx) improved physiological alertness, but did not affect performance on inhibitory capacity and working memory. Again, the differences in light properties, individual characteristics (i.e., whether habitual napper or not), and study paradigm make it difficult to directly compare the findings between these studies and the current one. In addition, the nature (i.e., type of task or task difficulty) of the task would also partly explain these inconsistencies. For instance, one study by Huiberts et al. (29) reported that the bright light did more benefit on the performance of difficult vs. easy level of working memory task as assessed with Backwards Digit-Span Task. Also, one recent study by Ru et al. (30) reported that reaction speed in the go/no-go task and Flanker task (only incongruent trials) was significantly enhanced with bright vs. dim light, but not in the PVT or the PVSAT. Combining the current findings suggested that bright blue-enriched white light could hold the potentials to enhance alertness performance and working memory in the early afternoon, especially for those who are temporarily midday nap deprived.

Some limitations of the current study need to be mentioned when drawing conclusions. Firstly, although participants were required not to spend too much time outside and keep their regular sleep–wake schedule on the experimental days, light history, night time sleep, and also diet for lunch prior to the laboratory study were not exactly followed in the current study, which might influence individual physical state and fatigue and confuse effects of light intervention. Second, the illuminance and color temperature did not match between normal indoor light condition and blue-enriched bright light condition, such that it makes less possible to disentangle the relative contribution of high illuminance or short wavelength spectrum to the current light-induced benefits on subjective state and performance. More refined light scenarios should be applied in future studies to identify which properties (illuminance or correlated color temperature) might be more powerful to counteract the post-lunch dip in subjective state and performance. Lastly, although not the interest in the present study, some individual differences [i.e., age (72), gender (73), and chronotype (74)] deserve to be explored in the future to test the generalization of the current findings.

## Conclusions

The current findings demonstrated that a habitual post-lunch nap deprivation would, to a larger extent, impair habitual nappers' subjective vigilance, mood, and performance in sustained attention and working memory task. Exposure to bright blue-enriched white light vs. normal indoor light could partially function as a strategy to counteract undesirable impairment on sleepiness, negative mood, and working memory caused by post-lunch nap deprivation among university students who were habitual napper, whereas no such benefits were found for sustained attention, response inhibition, and subjective positive mood. Yet, additional researches would be required when to transfer the current findings into practical working settings.

## Data Availability Statement

The raw data supporting the conclusions of this article will be made available by the authors, without undue reservation.

## Ethics Statement

The study involving human participants was reviewed and approved by Ethics Committee on Research involving Humans at South China Normal University. The participants provided their written informed consent to participate in this study.

## Author Contributions

TR conceptualized the study, responsible for project administration, and validated the data. YZ, XL, and LL curated the data. TR and GZ acquired funding for the study and supervised the study. YZ, QC, XL, and LL conducted all the investigations. YZ, QC, and TR performed the methodology. GZ gathered resources. QC was responsible for the software used in this study and responsible for the visualization. YZ and TR wrote the original draft. QC and TR reviewed and edited the manuscript. All authors contributed to the article and approved the submitted version.

## Conflict of Interest

The authors declare that the research was conducted in the absence of any commercial or financial relationships that could be construed as a potential conflict of interest.
